# Immunomodulatory activity of *Pleurotus pulmonarius* crude extract to human monocyte against *Cryptococcus neoformans*

**DOI:** 10.1186/s12906-025-04990-z

**Published:** 2025-07-09

**Authors:** Artittaya Arromsava, Siriporn Chuchawankul, Navaporn Worasilchai, Pornpimon Angkasekwinai, Kamolporn Amornsupak

**Affiliations:** 1 Program of Molecular Sciences in Medical Microbiology and Immunology, Faculty of Allied Health Sciences, Chulalongkorn University, Bangkok, 10330 Thailand; 2 Department of Transfusion Medicine and Clinical Microbiology, Faculty of Allied Health Sciences, Chulalongkorn University, Bangkok, 10330 Thailand; 3 Immunomodulation of Natural Products Research Unit, Chulalongkorn University, Bangkok, 10330 Thailand; 4 Research Unit of Medical Mycology Diagnosis, Chulalongkorn University, Bangkok, Thailand; 5 Department of Medical Technology, Faculty of Allied Health Sciences, Thammasat University, Pathum Thani, 12120 Thailand

**Keywords:** *Pleurotus pulmonarius*, Monocyte, Macrophage, *Cryptococcus neoformans*, Immunomodulator

## Abstract

**Background:**

Treatment of *Cryptococcus neoformans* infection is still challenging due to high side effects and acquired drug resistance. Eliminating fungal infection requires monocytes and macrophages, which are weakened by the non-protective type-2 immune response induced by the pathogen. *Pleurotus pulmonarius* (PP) is an edible mushroom rich in bioactive molecules, particularly β-glucan. This study aimed to investigate the immunomodulatory activities of PP crude extract on monocytes and macrophages against *C. neoformans.*

**Methods:**

PP was extracted using a hot aqueous method and precipitated with absolute ethanol. Glucan content was assessed by colorimetric assay. The cytotoxicity of PP crude extract was tested using an MTT assay. The immunomodulatory activities and preventive potential of PP crude extract for *C. neoformans* infection were investigated using human THP-1 monocyte and THP-1-derived macrophage models. Proinflammatory and anti-inflammatory cytokine production was evaluated using RT-qPCR and ELISA. Immune mechanisms related to *C. neoformans* clearance were studied, including phagocytosis, ROS production, macrophage polarization, and antifungal killing.

**Results:**

The result showed that PP crude extract contains α-glucan (14.72% w/w) and β-glucan (11.75% w/w). The immunomodulatory activities of PP crude extract on the expressions of *TNFA, IL1B, IL6,* and *MCP1/CCL2* were observed in THP-1 cells. This finding corresponds to the significant increase in TNF-α level measured by ELISA. Moreover, PP crude extract enhanced the release of TNF-α and IL-10 from THP-1-derived macrophages. Interestingly, pre-exposure to PP crude extract elicits preventive potential for controlling *C. neoformans* infection. A significant increase in type 1 cytokines was observed in *C. neoformans*-infected monocytes and macrophages. While *C. neoformans* induced M2 polarization, this phenotype was attenuated in PP-trained macrophages partly through *MCP1/CCL2* expression and CD206 M2 suppression. Moreover, 100 µg/mL of PP crude extract pretreatment could impair *C. neoformans* intracellular proliferation in THP-1-derived macrophages and upregulate ROS production.

**Conclusions:**

This study highlights the immunomodulatory potential of PP crude extract as a plant-based immunomodulator to enhance protective antimicrobial responses, particularly through ROS-mediated mechanisms of antifungal immunity*.* These findings support its potential application in developing natural therapeutic supplements, functional foods, and trained immunity-based strategies as alternative approaches to strengthening host defense against infections.

## Introduction

*Cryptococcus neoformans* is an opportunistic fungal pathogen classified as a critical priority on the World Health Organization’s fungal priority pathogens list [1]. This fungus significantly influences morbidity and mortality rates worldwide, particularly among immunocompromised individuals, including people with HIV/AIDS, organ transplant recipients, and those undergoing chemotherapy [2, 3]. The lack of available vaccines and the limited number of effective antifungal medications, which have side effects and lead to acquired drug resistance, make such life-threatening infections a more serious global health challenge [4–6].

The type-1 immune response, which involves monocytes and macrophages, serves as a crucial first line of defense against fungal infections. During the early stages of a *C. neoformans* infection, monocytes are recruited to the infection site, where they play a vital role in eliminating the fungus. They achieve this through processes such as phagocytosis, the secretion of pro-inflammatory cytokines, and the presentation of antigens to T lymphocytes. However, *C. neoformans* has developed several virulence factors to evade the host immune response. Notably, it can damage immune cells using specific toxins and promote a non-protective type 2 immune response along with M2 macrophage polarization [7]. Furthermore, this fungus can survive inside phagocytes, effectively using them as a Trojan horse to disseminate from the lungs to the central nervous system, which can lead to fatal cryptococcal meningitis [8]. Therefore, dysfunction of monocytes and macrophages primarily drives the onset and progression of the disease.

The diverse range of bioactive compounds and nutraceutical components found in edible mushrooms has ignited widespread interest as both complementary medicine and functional food. These include β-glucans, polysaccharides, polyphenols, and other phytochemicals, all of which have been linked to various potential health benefits, including immune support [9, 10].

β-glucans are promising immunomodulatory polysaccharides composed of d-glucose units. These are naturally found in algae, oats and barley, probiotic bacteria, and fungi, including yeast and edible mushrooms [10]. β−1,3/1,6-D-glucan is recognized as one of the main pathogen-associated molecular patterns. It binds to Dectin-1, a well-characterized pattern recognition receptor (PRR) involved in antifungal immunity via the Syk-NF-κB signaling cascade [11, 12]. Previous studies revealed that β-glucan-containing exopolysaccharides from *Auricularia auricular* could modulate macrophage immune response against *C. neoformans* infection by enhancing phagocytosis and killing activities [13]. Therefore, immunotherapy that utilizes natural substances to activate innate immunity represents a compelling strategy for addressing fungal infections.

*Pleurotus pulmonarius* (PP) is a domestically cultivated edible mushroom. PP shows promise as a medical mushroom due to its abundance of β−1,3/1,6-D-glucan and its potential role as an immunomodulator [14]. This study aimed to investigate the immunomodulatory effects of PP crude extract on the immune responses of monocytes and macrophages against *C. neoformans* infection. We assessed important factors, including cytokine production, cellular polarization, phagocytosis, and fungicidal activities, utilizing THP-1 monocytes and THP-1-derived macrophages as models. The study proposes PP as a potential plant-based immunomodulator to improve innate immune responses and help prevent microbial infections, particularly those caused by *C. neoformans*. This alternative treatment may be beneficial for immunocompromised individuals while reducing the risk of antibiotic resistance and the adverse effects associated with antifungal drugs.

## Methods

### Ethics approval and biosafety approval

This study was conducted in accordance with the ethical principles outlined in the Declaration of Helsinki. Human serum was collected from healthy volunteers who provided written informed consent. The study protocol received approval from the Research Ethics Review Committee for Human Research Participants, Group 1, at Chulalongkorn University (COA No. 041/67). The authors confirm that all experiments were carried out following the relevant institutional guidelines and regulations. All experiments were conducted at biosafety level 2 and were approved by the Institutional Biosafety Committee, Faculty of Allied Health Sciences, Chulalongkorn University, under Certificate of Approval No. AHS-IBC 008/2022.

### PP crude extract preparation

The mushroom was identified by Assist. Prof. Dr. Jittra Piapukiew, Department of Botany, Faculty of Science, Chulalongkorn University (Voucher specimen: MZ395974), Bangkok, Thailand. *P. pulmonarius* was extracted through a hot water method to obtain soluble polysaccharides and precipitation with absolute ethanol, based on previous studies with some modifications [15, 16]. Briefly, dried mushrooms were ground into a fine powder. A total of 100 mg of the mushroom powder was extracted with 1,000 mL of distilled water at a ratio of 1:10 (w/v) at a temperature of 95 ± 5 °C for 1 h. The mixture was then filtered to separate the mushroom residue. The residue was extracted two additional times, and the collected supernatant was centrifuged. Subsequently, the resulting aqueous solution was first filtered through 11 µm Whatman No. 1 filter paper, followed by further filtration using a 0.2 µm syringe filter to eliminate microbial contamination. The solution was then precipitated with 80% (v/v) ethanol at 4 °C for 18 h. Following this, the mixture was centrifuged at 4 °C, 4,500 rpm for 10 min, and the pellet was washed with absolute ethanol. Finally, to obtain the PP crude extract, the ethanol was removed by incubating the pellet at 70 °C. The crude extract was then dissolved in sterile phosphate-buffered saline (PBS) to create a stock solution, which was stored at −80 °C.

### β-glucan measurement

The glucan component of PP crude extract was quantified by colorimetric assay using the β-glucan assay kit (Megazyme, USA), according to the manufacturer’s instructions. Glucan content was calculated using a Mega-Calc™ data calculator.

### Bacterial and lipopolysaccharide (LPS) contamination testing

The PP crude extract solution (50 mg/mL, 100 µL) was added to the nutrient broth and incubated at 37 °C for 72 h to measure microbial growth. To assess potential LPS contamination, THP-1-derived macrophages (2 X 10^5^ cells/well) were stimulated with 500 µg/mL of PP crude extract, both with and without 10 µg/mL of polymyxin B, for 16 h. Afterward, the culture medium was collected to measure TNF-α level using an ELISA kit (Sino Biological, China). As a control, THP-1-derived macrophages were treated with 10 ng/mL of LPS, with and without 10 µg/mL of polymyxin B.

### Cell line

The THP-1 monocyte cell line was kindly provided by Assoc. Prof. Dr. Panan Ratthawongjirakul (Faculty of Allied Health Sciences, Chulalongkorn University, Thailand). The cells were cultured in Roswell Park Memorial Institute 1640 Medium (RPMI) (Cytiva, USA) supplemented with 10% (v/v) fetal bovine serum (Invitrogen, USA), 1% (v/v), antibiotic, and 1% Glutamax (Invitrogen, USA). The culture was maintained at 37 °C in 5% CO_2_. Differentiation of THP-1 monocytes into THP-1-derived macrophages was performed as previously described [17], with some modifications. THP-1 monocytes (2 X 10^5^ cells/mL) were seeded in a poly-L-lysine-coated 24-well plate. The monocytes were stimulated with 50 nM Phorbol 12-myristate 13-acetate (PMA) (Sigma-Aldrich, USA) for 48 h. Following stimulation, the activated cells were washed with RPMI medium and then rested in complete medium for an additional 48 h. Changes in cell morphology were observed under a microscope. Adherent cells were detached using TrypLE™ Express Enzyme (Thermo Fisher Scientific, USA), and subsequently stained with a FITC-labelled anti-CD11b antibody (Biolegend, USA). The expression of CD11b was quantified using a flow cytometer (Beckman Coulter, USA) to assess macrophage differentiation.

### Culture of *C. neoformans* and opsonization

The *C. neoformans* var. grubii (serotype A) H99 strain was kindly provided by Dr. Navaporn Worasilchai (Faculty of Allied Health Sciences, Chulalongkorn University, Thailand). The yeast was cultured on Sabouraud dextrose agar (SDA) (Difco, USA) at 25 °C for 48 h. Following this, a single colony was inoculated into Sabouraud dextrose broth (Difco, USA) and incubated at 30 °C with shaking at 230 rpm for 24 h. Next, the yeast cultures were centrifuged at 3,500 rpm for 5 min, washed with PBS, and then resuspended in RPMI medium. Prior to co-culture, *C. neoformans* was opsonized using fresh human serum. The serum was obtained by centrifuging clotted whole blood at 4 °C, 3,500 rpm for 5 min, immediately after the clot had formed. The separated fresh human serum was then mixed with the yeast cell suspension at a ratio of 40% (v/v). The mixture was opsonized at 37 °C with shaking at 120 rpm for 1 h. After this, opsonized *C. neoformans* were centrifuged, washed with PBS, and resuspended in complete medium. The viability of the opsonized *C. neoformans* was assessed using trypan blue staining. Yeasts with a cell viability greater than 95% were counted, and the concentration was adjusted to achieve a multiplicity of infection (MOI) of 1:5.

### Cell cytotoxicity of PP crude extract

THP-1 monocytes (2 X 10^4^ cells/50 µL) were seeded in a 96-well plate and stimulated with 50 µL of PP crude extract at varying concentrations for 24 h. Afterwards, 5 µg/mL of 3-(4,5-Dimethyl-2-thiazolyl)−2,5-diphenyl-2H-tetrazolium bromide (MTT) reagent (Sigma-Aldrich, USA) was added and incubated at 37 °C for 4 h in the dark. After that, 100 µL of 10% (v/v) Sodium Dodecyl Sulfate (SDS) was added to dissolve the crystal. The absorbance was measured at 570 nm using a Synergy H5 microplate reader (BioTek, USA). Cell viability was calculated relative to the non-treated control as follows:

Cell viability (%) = [OD_570_(PP-treated groups)/OD_570_(non-treated control)] X 100.

### Immunomodulatory of PP crude extract on THP-1 monocytes and THP-1 derived macrophages

THP-1 monocytes (5 X 10^5^ cells/mL) were stimulated with 100 and 500 µg/mL of PP crude extract for designated periods. After 12 h of stimulation, the cells were harvested for RNA extraction, followed by cDNA synthesis. The expression levels of the *TNFA, IL1B, IL6, MCP1/CCL2,* and *IL10* genes were analyzed using quantitative reverse transcription PCR (RT-qPCR). The relative gene expression in the treated groups was normalized against the non-treated control. Additionally, the culture medium was collected after 16 h of stimulation to measure the cytokine levels of TNF-α and IL-10 using ELISA kits (Sino Biological, China). To evaluate the effect of PP crude extract on cytokine release from macrophages, THP-1-derived macrophages (2 X 10^5^ cells/mL) were activated with 5, 100, and 500 µg/mL of PP crude extract for 16 h, after which the culture medium was collected for cytokine measurement.

### Quantitative Real-Time PCR

THP-1 monocytes and THP-1-derived macrophages were harvested at the specified time points. Total RNA was extracted using the GENEzol™ reagent (Geneaid, Taiwan) according to the manufacturer’s protocol. The quantity of RNA was measured by a NanoDrop™ (Thermo Fisher Scientific, USA). cDNA was synthesized using the RevertAid First Strand cDNA Synthesis Kit (Thermo Fisher Scientific, US). RT-qPCR was performed using the iTaq Universal SYBR Green Supermix (Biorad, USA). Then, the fluorescence signal was quantified using the QuantStudio™ 5 Real-Time PCR System (Thermo Fisher Scientific, USA). The quantification cycle (Cq) of the targeted genes was compared to the *ATCB* gene. The relative gene expression (fold change) of the test group was normalised against the negative control using the 2^−ΔΔct^ method. All reported data represent a minimum of three independent experiments, and the gene-specific primers used are listed in Table 1.

**Table 1:** List of primer sequences

Target gene	Sequence (5’—> 3’)
***MCP1/CCL2***	F’ CAAACTGAAGCTCGCACTCTC
	R’ CCTCTGCACTGAGATCTTCCT
***TGM2***	F’ GACAACAACTACGGGGACGG
	R’ ATGGGCCGAGTTGTAGTTGG
***TNFA***	F’ CAGGCGGTGCTTGTTCCTCA
	R’ GGCTTGTCACTCGGGGTTCG
***IL1B***	F’ GCTGATGGCCCTAAACAGATGA
	R’ TCCTGGAAGGTCTGTGGGCA
***IL6***	F’ AGTGAGGAACAAGCCAGAGC
	R’ AGCTGCGCAGAATGAGATGA
***IL10***	F’ GACCCAGACATCAAGGCGCA
	R’ ATTCTTCACCTGCTCCACGGC
***ATCB***	F’ AGAAAATCTGGCACCACACCT
	R’ GATAGCACAGCCTGGATAGCAA

### Preventive effect of PP crude extract on *C. neoformans* infection in THP-1 monocytes and THP-1 derived macrophages

The THP-1 in vitro model was established to investigate the role of PP crude extract in modulating the immune responses of human monocytes and macrophages, which are crucial in controlling *C. neoformans* infection. Two experimental models were adapted based on previous studies [18, 19]. In the first model, designed to examine the monocyte response, THP-1 monocytes (5 X 10^5^ cells/mL) were seeded into a 6-well plate and pretreated with either 100 µg/mL (designated as tPP-100) or 500 µg/mL (designated as tPP-500) of PP crude extract for 24 h. THP-1 monocytes cultured in complete medium without pretreatment served as a “non-trained control” (NT). After pretreatment, the THP-1 monocytes, now referred to as “trained monocytes”, were washed with RPMI medium and rested for 48 h. Following this resting phase, the trained monocytes were challenged with opsonized *C. neoformans* (MOI 1:5) for 24 h. Subsequently, the expression levels of the *TNFA, IL1B,* and *IL6* genes were analyzed after restimulation.

The second model focused on macrophage response. THP-1 monocytes were pretreated with PP crude extract as described above, then activated with 50 nM PMA to differentiate into macrophages, referred to as “trained macrophages.” These trained macrophages were challenged with opsonized *C. neoformans* (MOI 1:5) for 2 h. After that, the extracellular yeasts were removed, and the infected cells were cultured for an additional 16 h. The expression levels of the *TNF-α*, *IL-1β*, and *IL-10* genes were quantified 18 h post-infection. Furthermore, the antifungal activities involved in eliminating *C. neoformans*, including phagocytic activity, reactive oxygen species (ROS) generation, and colony-forming unit (CFU) inhibition assay, were assessed.

### Macrophage polarization and M2 phenotype suppression

*C. neoformans* typically induces a Type 2 immune response characterized by the presence of IL-4, which promotes macrophage polarization toward the M2 phenotype [7]. To investigate whether PP crude extract modulates this polarization, trained macrophages (2 X 10^5^ cells) were co-cultured with opsonized *C. neoformans* for 2 h*.* Following co-culture, extracellular yeasts were removed. The infected macrophages were then cultured for 24 h, with or without the addition of 20 ng/mL of rhIL4 (Immunotools, Germany). To assess surface marker expression, the infected macrophages were washed with ice-cold PBS and detached using the TrypLE™ Express Enzyme. The cells were then washed twice with an ice-cold washing buffer and then blocked with 2% BSA in PBS on ice for 30 min. Subsequently, the cells were stained with fluorochrome-conjugated antibodies: FITC anti-human CD11b (cat. 301,330), PE/Cyanine anti-human CD80 (cat. 305,218), and APC anti-human CD206 (MMR) (cat. 321,110), all sourced from BioLegend (USA). After staining, cells were washed twice and resuspended in ice-cold buffer before flow cytometric analysis. CD11b⁺ macrophages were gated, and the expression of CD80 and CD206 was quantified to assess M1/M2 polarization status. In parallel, infected macrophages were harvested at 18 h post-infection for total RNA extraction. Gene expression levels of *MCP1/CCL2* (M1 marker) and *TGM2* (M2 marker) were measured using RT-qPCR. The percentages of CD80⁺ and CD206⁺ cells, along with relative gene expression levels, were compared between trained and non-trained macrophages. As polarization controls, THP-1-derived macrophages stimulated with 20 ng/mL rhIFN-γ plus 10 ng/mL LPS (for M1) or with 20 ng/mL rhIL-4 (for M2) were used.

#### Phagocytosis and CFU inhibition assays

Briefly, trained macrophages (2 X 10^5^ cells) were infected with opsonized *C. neoformans* for either 2 h or 24 h. After incubation, unbound yeast cells were removed, and the macrophages were lysed with 0.05% SDS to release the intracellular yeasts. The lysates were resuspended in PBS, plated on SDA, and incubated at 30 °C for 48–72 h before counting the colonies. In parallel, the infected macrophages were cultured for up to 24 h post-infection. Both intracellular and extracellular cryptococci were retrieved. CFU assays were performed according to established protocols. The CFU count at 2 h post-infection was used to calculate the phagocytic index. The intracellular proliferation rate (IPR) of *C. neoformans* at 24 h post-infection was determined using the formula: [(CFU at 24 h – CFU at 2 h)/CFU at 2 h] [20].

#### Reactive oxygen species (ROS) assay

The ROS production was assessed using a Dihydroethidium assay kit (Abcam, USA). In brief, macrophages infected with *C. neoformans* were collected at 2 h post-infection. The cell pellet was washed and resuspended in cell-based assay buffer, followed by centrifugation at 400 × g to remove the residual medium. The cells were then stained with ROS staining buffer and incubated at 37 °C for 30 min in the dark. After incubation, the staining buffer was removed, and the cells were resuspended in 100 µL of assay buffer. Fluorescence intensity was subsequently measured using the flow cytometer to quantify ROS levels.

#### Statistical analysis

Data were presented as mean ± SEM. Statistical significance was calculated using an independent t-test and one-way ANOVA with Turkey post hoc or Dunnett’s test through SPSS software. A *P*-value ≤ 0.05 was considered statistically significant.

## Results

### Glucan component, LPS contamination of PP crude extract

**Fig. 1:**
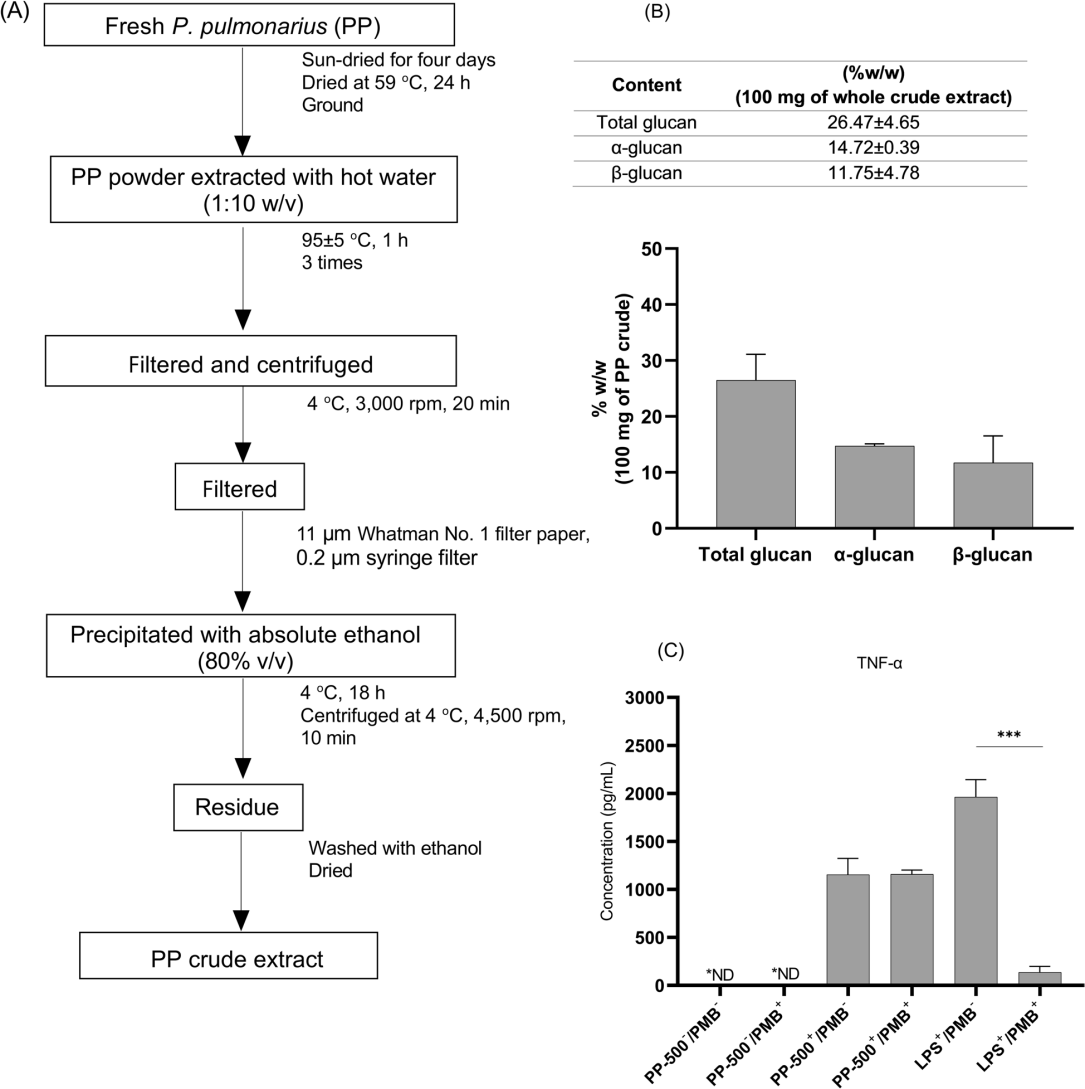
PP crude extract preparation (**a**), an evaluation of the glucan component in PP crude extract (**b**), and LPS contamination monitoring (**c**). PP crude extract was prepared by hot water extraction and precipitation methods. Using an enzymatic approach, total glucan and α-glucan were directly measured, whereas β-glucan was estimated using an equation. LPS contamination was indirectly determined by using polymyxin B (PMB). First, THP-1 monocytes were activated with 50 nM PMA to induce macrophage differentiation. After that, the macrophages were activated with PP crude extract with or without PMB for 16 h. TNF-α production was evaluated by ELISA assay. The results are presented as mean ± SEM of two independent experiments. *P-values* (***) ≤ 0.001

Glucan is a natural polysaccharide component in the cell walls of bacteria and fungi. Previous studies revealed the immunomodulatory function of natural α- and β-glucans in mushrooms against microbial infections [13, 21]. PP crude extract was prepared by hot water extraction and precipitation method to obtain the water-soluble polysaccharides (Fig. 1A). The percentage of soluble glucans, including α-glucan and β-glucan were 14.72% (w/w) and 11.75% (w/w), respectively (Fig. 1B). To ensure that the immunomodulatory effects of PP crude extract were not due to endotoxin contamination, we assessed the presence of LPS during the extraction process. Polymyxin B, a cationic antibiotic that binds and neutralizes the lipid A component of LPS, was used to evaluate potential contamination [22]. No detectable LPS contamination was found in the PP crude extract at concentrations up to 500 µg/mL, as indicated by the unchanged TNF-α levels in THP-1-derived macrophages treated with PP crude extract, with or without 10 µg/mL polymyxin B (Fig. 1C). These results confirm that the immunomodulatory activity of PP extract is attributed to its bioactive compounds rather than LPS contamination.

### Cell cytotoxicity of PP crude extract on THP-1 monocytes

**Fig. 2:**
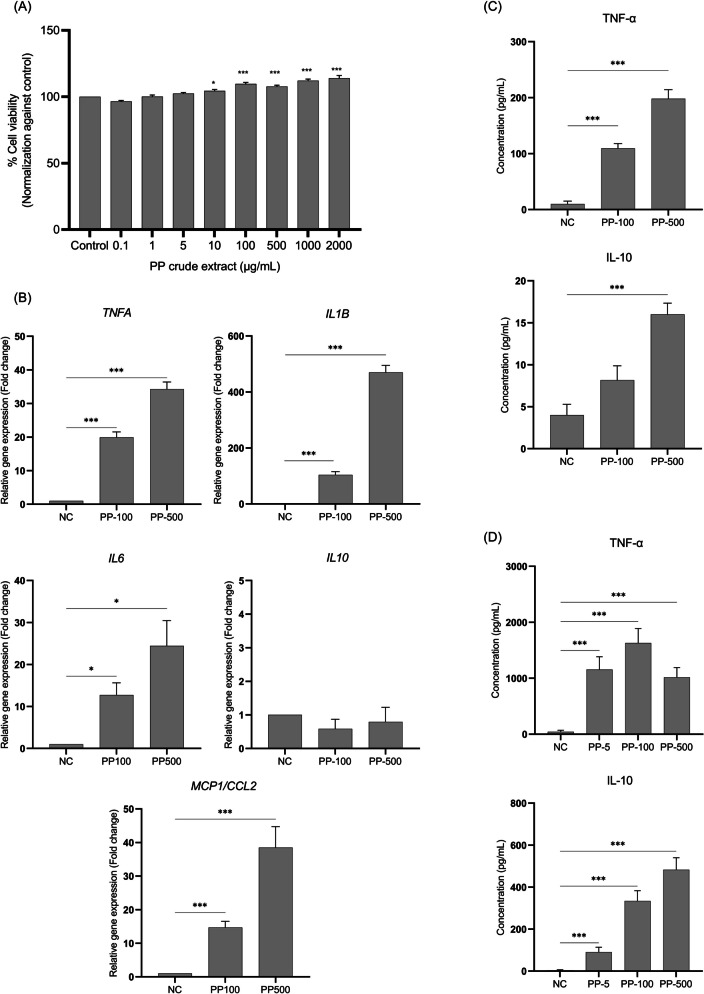
Cell cytotoxicity and immunomodulatory effect of PP crude extract. THP-1 monocytes were stimulated with PP crude extract for 24 h. After that, the cytotoxicity of PP crude extract was evaluated using the MTT assay. %Cell viability was normalized against non-treated control groups (**a**). The immunomodulatory activities of PP crude extract on THP-1 monocytes and THP-1-derived macrophages were studied using RT-qPCR and ELISA. THP-1 monocytes were treated with 100 µg/mL and 500 µg/mL of PP crude extract. After 12 h and 16 h of stimulation, cells were harvested for gene expression studies (**b**) and culture medium was collected for cytokine measurement (**c**), respectively. mRNA expression was analyzed as a relative expression to *ATCB*. The immunomodulatory effect of PP crude extract on cytokine release from THP-1-derived macrophages was assessed using ELISA at a specified time (**d**). The results are presented as mean ± SEM of three independent experiments. A statistical analysis was performed: the one-way ANOVA with Dunnett’s test and an independent t-test. *P-values* (*) ≤ 0.05, and (***) ≤ 0.001

To investigate the cytotoxicity of PP crude extract on THP-1 monocytes and find the appropriate dose. THP-1 monocytes were treated with PP crude extract for 24 h. Subsequently, an MTT assay was performed. The result showed that no cell cytotoxicity was observed in THP-1 monocytes after 24 h of incubation with PP crude extract up to 2,000 µg/mL. Furthermore, cell viability was significantly increased to 109.70 ± 5.14%, 107.70 ± 4.22%, 112.10 ± 5.03%, and 114.00 ± 8.61% in PP crude extract treatment at 100, 500, 1,000, and 2,000 µg/mL, respectively (Fig. 2A).

### Immunomodulatory effects of PP crude extract on THP-1 monocytes and THP-1 derived macrophages

To assess the direct impact of PP crude extract on cytokine production in THP-1 monocytes, the expression of proinflammatory and anti-inflammatory cytokines in PP-treated cells after 12 h and 16 h was determined using RT-qPCR and ELISA assay, respectively. The findings revealed that PP crude extract significantly upregulated the expressions of a broader spectrum of proinflammatory cytokines in THP-1 monocytes, including *TNFA* and *IL1B, IL6* and *MCP1/CCL2,* in a dose-dependent manner (Fig. 2B)*.* Furthermore, the release of TNF-α significantly increased at 16 h after activation with 100 and 500 µg/mL of PP crude extract, reaching levels of 109.3 and 198.0 pg/mL, respectively (Fig. 2C-1). However, only the concentration of 500 µg/mL of PP crude extract notably enhanced IL-10 production in THP-1 monocytes (Fig. 2C-2). Interestingly, the immunomodulatory impact of PP crude extract is more pronounced in THP-1-derived macrophages compared to THP-1 monocytes (Fig. 2C, D). The TNF-α and IL-10 production in THP-1-derived macrophages exhibited statistical significance at a minimum concentration of 5 µg/mL of PP crude extract. (Fig. 2D). These findings suggest that PP crude extract possesses the potential to elicit both proinflammatory and anti-inflammatory cytokines in a dose-dependent and cell-type-specific manner.

### Pretreatment with PP crude extract promotes proinflammatory cytokine production in THP-1 monocytes and THP-1-derived macrophages after *C. neoformans* infection

**Fig. 3:**
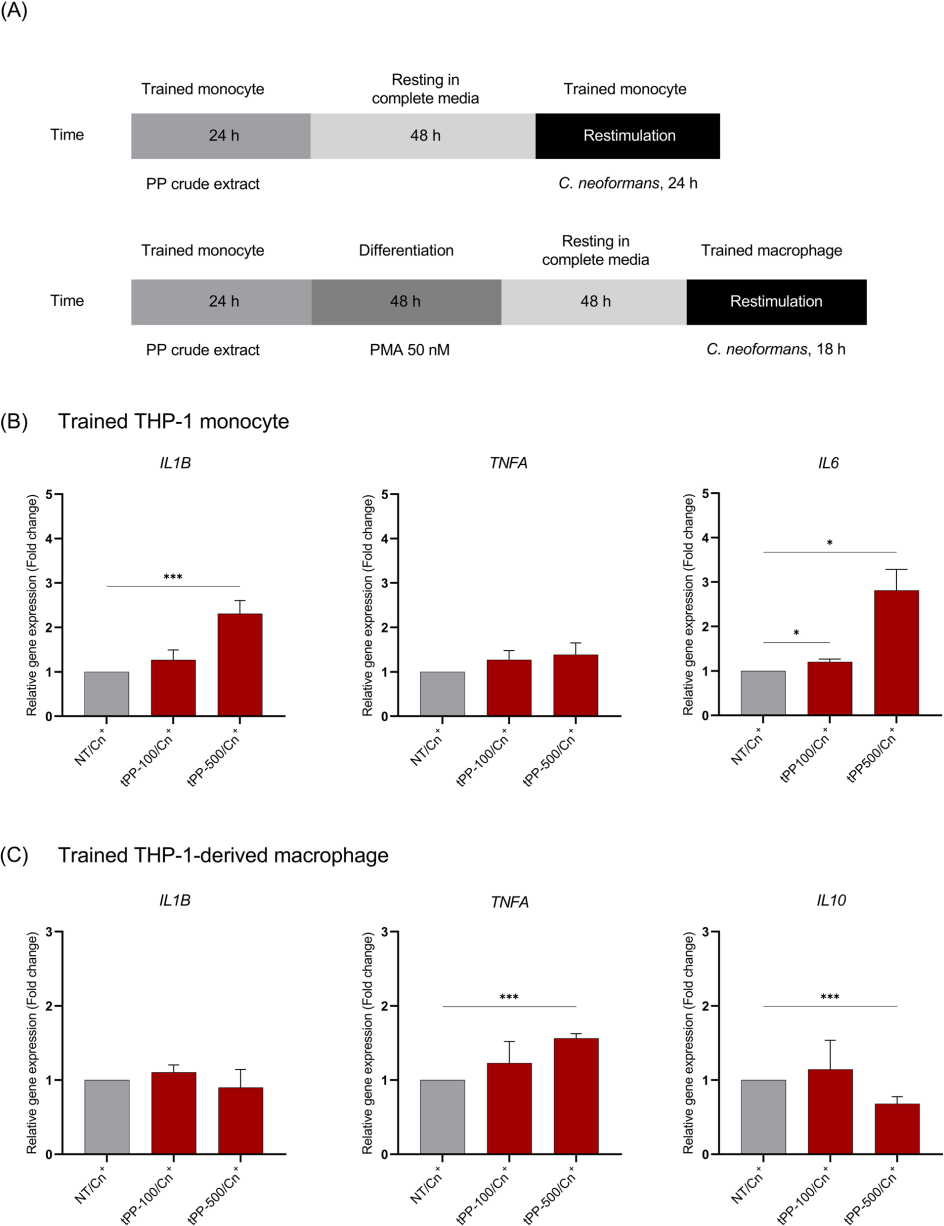
Preventive potential of PP crude extract on cytokine production in THP-1 monocytes and THP-1 derived macrophages. THP-1-preventive models were established (**a**). Briefly, THP-1 monocytes were either pre-exposed to 100 µg/mL or 500 µg/mL of PP crude extract for 24 h. THP-1 monocyte model, PP-treated cells were rested for 48 h, followed by restimulation with opsonized *C. neoformans* (MOI 1:5) for 24 h. Relative gene expressions of *IL1B, TNFA*, and *IL6* were analyzed to verify the effect of PP crude extract on secondary immune response to *C. neoformans* (**b**). THP-1-derived macrophage model, trained THP-1 monocytes were initially stimulated with 50 nM PMA for 48 h to induce macrophage differentiation, followed by resting for 48 h. After differentiation into macrophages, the trained THP-1-derived macrophages were restimulated with opsonized *C. neoformans* for 18 h. Subsequently, gene expression analysis was performed to assess the gene expression levels of *IL1B*, *TNFA*, and *IL10* (**c**). The results are presented as Mean ± SEM of three independent experiments. A statistical analysis was performed using an independent t-test. *P-values* (*) ≤ 0.05 and (***) ≤ 0.001

It is increasingly acknowledged that when exposed to certain stimuli, innate immune cells, such as monocytes and macrophages, can evoke non-specific protective effects and adeptly modulate their response to subsequent insults [23]. To explore whether pre-exposure of monocytes to PP crude extract, containing β−1,3/1,6-D-glucans, the same component found in the yeast cell wall, could elicit cross-protective effects against fungal infection, preventive models utilizing THP-1 monocytes and *C. neoformans* infection were established according to the schematic diagram (Fig. 3A-1). THP-1 monocytes were pretreated with 100 and 500 µg/mL of PP crude extract for 24 h, followed by resting in complete media for 48 h. The pretreated or trained cells were subsequently challenged with opsonized *C. neoformans* (MOI 1:5). Gene expression levels related to non-specific protective effects were examined at 24 h post-restimulation. The results revealed that pre-exposure to 100 µg/mL of PP crude extract could significantly increase the expression level of *IL6* gene in THP-1 monocytes after *C. neoformans* infection compared to the untrained group (Fig. 3B-3). Moreover, exposure to 500 µg/mL of PP crude extract significantly increased the expression levels of *IL1B* and *IL6* genes in *C. neoformans*-infected cells (Fig. 3B-1,3). These findings suggest that PP could potentially enhance the production of proinflammatory cytokines in response to subsequent infection.

Next, to further ascertain whether the immunomodulatory effect persists following the differentiation of trained monocytes into macrophages, the trained THP-1 monocytes were treated with PMA to induce macrophage differentiation before *C. neoformans* restimulation (Fig. 3A-2). In contrast to trained monocytes, expression of the *IL1B* gene was not upregulated in trained THP-1-derived macrophages (Fig. 3C-1). Interestingly, *TNFA* gene expression was significantly increased, while *IL10* was significantly downregulated in the macrophages trained with 500 µg/mL PP crude extract (Fig. 3C-2,3). These findings indicate that pre-exposure to PP crude extract could promote a sustained proinflammatory response in macrophages against *C. neoformans* infection.

### Pretreatment with PP crude extract enhances M1 macrophage polarization and M2 phenotype suppression

**Fig. 4:**
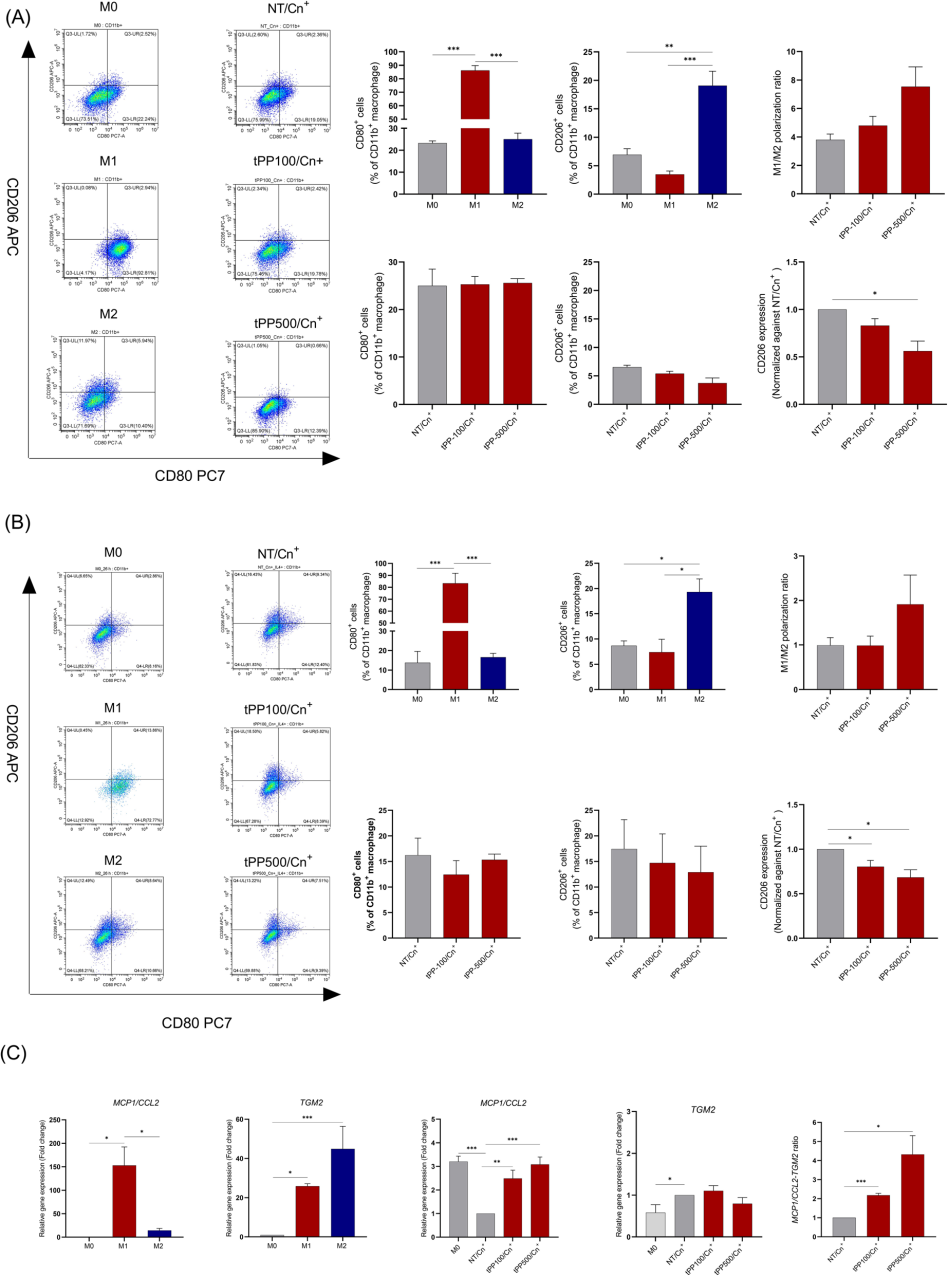
Preventive potential of PP crude extract on macrophage polarization and M2 phenotype suppression. Trained THP-1 monocytes were activated with 50 nM PMA to induce macrophage differentiation. The surface marker expression of CD80 (M1 marker) and CD206 (M2 marker) on trained macrophages were measured using flow cytometry after restimulation with opsonized *C. neoformans* (MOI 1:5) in the absence (**a**) or presence of 20 ng/mL of IL-4 cytokine (**b**). The M1/M2 polarization ratio was calculated. In addition, CD206 expression in trained macrophages was normalized to that of non-trained macrophages after infection with *C. neoformans* (a,b). In the control condition, the M0 macrophages were activated with 20 ng/mL of rhIFN-γ plus 10 ng/mL of LPS to induce M1 polarization, while 20 ng/mL of rhIL-4 was used to induce the M2 phenotype. Moreover, trained THP-1-derived macrophages were restimulated with opsonized *C. neoformans*, and gene expression levels of *MCP1/CCL2* and *TGM2* were analyzed at 18 h post-infection. The *MCP1*/*CCL2*-to-*TGM2* ratio was subsequently calculated to assess the polarization state (**c**). The results are presented as Mean ± SEM of three independent experiments. A statistical analysis was performed using an independent t-test and one-way ANOVA with Turkey post-hoc or Dunnett’s test. *P-values* (*) ≤ 0.05, (**) ≤ 0.01 and (***) ≤ 0.001

**Fig. 5:**
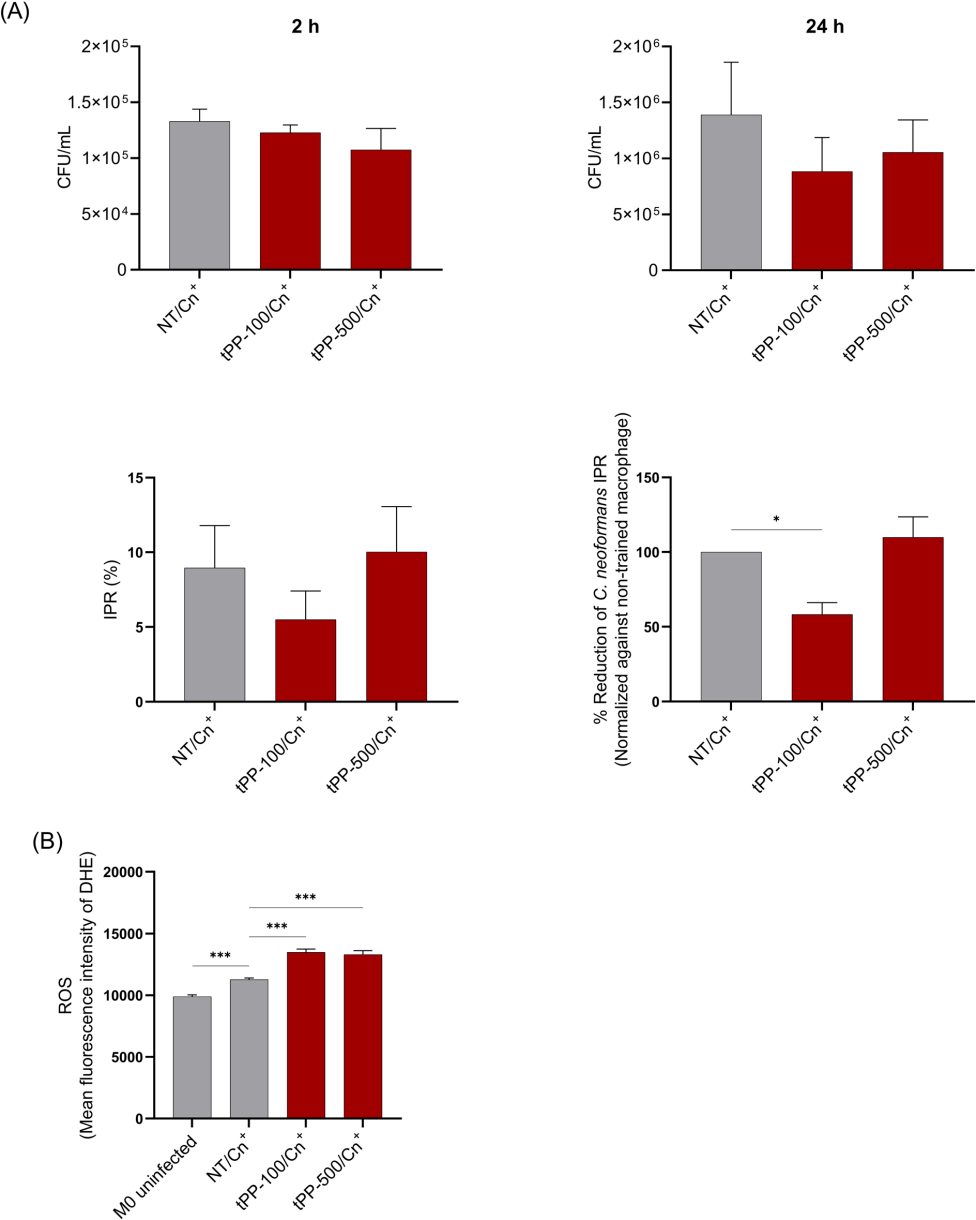
Preventive potential of PP crude extract on *C. neoformans* clearance by trained THP-1-derived macrophage. Trained THP-1-derived macrophages were co-cultured with opsonized *C. neoformans* (MOI 1:5) for 2 h, then the extracellular yeast was removed. Infected macrophage was lysed at 2 h, and 24 h post-infection to determine the phagocytosis index (**a**), and *C. neoformans* intracellular proliferation rate using CFU assay (**a**). % Reduction of intracellular proliferation rate was calculated by normalizing against non-trained macrophage (**a**). ROS production was determined at 2 h post-infection using flow cytometry, and the MFI of trained cells was compared with non-trained groups (**b**). Phagocytosis index, %IPR, % reduction of *C. neoformans* IPR, and MFI of ROS are presented as Mean ± SEM of two or three independent experiments. A statistical analysis was performed using the independent t-test. *P-values* (*) ≤ 0.05, and (***) ≤ 0.001

*C. neoformans* induce M2 macrophage polarization, which is one of the pathogen’s strategies for evading host immune elimination and disseminating to the central nervous system [24]. Therefore, we aimed to determine the effect of PP crude extract on macrophage polarization. CD80 (M1 marker) and CD206 (M2 marker) were evaluated using flow cytometry. The results revealed a significant decrease in CD206 expression and an increase in the M1/M2 polarization ratio in *C. neoformans*-infected macrophages that were pre-exposed to 500 µg/mL PP crude extract (Fig. 4A). Moreover, to recapitulate the suppressive microenvironment of *C. neoformans* infection which contains Th2 cytokines including IL-4, IL-5, and IL-13, promoting M2 polarization and repolarization of M1 macrophages into those with M2 phenotype [25, 26], the polarization of macrophages after infected with *C. neoformans* together with IL-4 cytokine was studied. In the non-trained group, the results showed that IL-4 slightly suppressed CD80 expression (from 25.0% to 16.2%) and markedly increased CD206 expression (from 6.5% to 17.4%) in *C. neoformans*-infected macrophages (Fig. 4B-2,3). Interestingly, the increase in M2 marker was significantly decreased in the infected macrophages pre-exposed to PP crude extract (Fig. 4B-7). Furthermore, the M1 marker, *MCP1/CCL2* and the M2 marker, *TGM2* in the infected macrophages were quantified by RT-qPCR. The results revealed that *MCP1/CCL2* expression was suppressed by *C. neoformans* (Fig. 4C-1)*,* which was significantly alleviated in the infected macrophages pre-exposed to PP crude extract (Fig. 4C-3). M1 phenotype-like cells were detected in trained macrophages, as suggested by the notable increase in the *MCP1/CCL2*-to-*TGM2* ratio in the PP crude extract-trained groups compared to the non-trained group with statistical significance (Fig. 4C-5). These findings suggest that pretreatment with PP crude extract may promote the M1 phenotype and suppress the M2 phenotype in *C. neoformans*-infected macrophages in a dose-dependent manner.

### Pretreatment with PP crude extract strengthens *C. neoformans* clearance by THP-1-derived macrophages

The main roles of macrophages in eliminating microorganisms include phagocytosis and pathogen destruction through the generation of an oxidative burst, and ROS [27]. However, *C. neoformans* employs various strategies to evade macrophage killing. Therefore, we investigated whether pretreatment with PP crude extract could enhance antifungal activity, including phagocytosis, killing activity, and ROS production against *C. neoformans.* Trained monocytes were differentiated into macrophages and restimulated with opsonized *C. neoformans*, as described earlier. CFU assays were conducted. The CFU counts at 2 h and 24 h post-infection were utilized to determine the phagocytic index and the intracellular proliferation rate (IPR). The data showed that pre-exposure to 500 µg/mL of PP crude extract tended to reduce phagocytosis at 2 h post-infection (Fig. 5A-1). Moreover, a decrease in CFU was observed in PP-trained macrophages at 24 h post-infection compared to the non-trained group (Fig. 5A-2). Furthermore, a notable reduction in the IPR was observed in macrophages trained with 100 µg/mL PP crude extract, significantly declining to 58% compared to non-trained cells (Fig. 5A-4). Additionally, ROS production in THP-1-derived macrophages was assessed after co-culturing with *C. neoformans* for 2 h. The results revealed that pretreatment with 100 µg/mL and 500 µg/mL of PP crude extract enhanced ROS production against *C. neoformans* infection (Fig. 5B). These findings imply that pre-exposure to PP crude extract could strengthen the clearance of *C. neoformans* in THP-1-derived macrophages, partly through the ROS production.

## Discussion

Plant-based immunotherapy has been a focus for decades due to its safety and potential therapeutic benefits. *P. pulmonarius* (PP), the Phoenix oyster mushroom, is a widely cultivated edible higher fungus used in food and folk medicine [14]. PP contains various nutraceutical compounds, such as β−1,3/1,6-D-glucan, a water-soluble polysaccharide akin to those found in yeast and fungal cell walls with antimicrobial activity [13]. However, the immunomodulatory potential of PP’s polysaccharides, especially in *C. neoformans* infection, remains untapped. This study aims to reveal a new therapeutic role of PP as a potent immunomodulator and to investigate its mechanisms and potential use as a natural preventive supplement for microbial infection.

Type 1 immune response by monocytes and macrophages is essential for eliminating microbial infection. Nevertheless, *C. neoformans* overcomes host immune responses through various strategies, including the induction of non-protective M2 macrophage polarization and using a monocyte/macrophage as a reservoir host, resulting in fungal dissemination to the central nervous system [8]. Antifungal drug therapy has high side effects with an increased incidence of acquired drug resistance [4–6]. Moreover, the incidence of such infection was highest in immunocompromised patients, particularly HIV-infected patients with a defective immune response [2, 3]. In this study, water-soluble polysaccharides from PP were extracted by the hot water technique, and glucan components, including α-glucans and β-glucans, were measured. PP crude extract comprises 11.75% (w/w) β-glucans and demonstrated no cytotoxic effects at concentrations up to 2,000 µg/mL compared with untreated control cells. The immunomodulatory effect of PP crude extract on proinflammatory and anti-inflammatory cytokine production in human THP-1 monocytes and THP-1-derived macrophages after 12 h and 16 h were assessed by RT-qPCR and ELISA, respectively. Our results demonstrated that PP crude extract had potential as an immunomodulator by strongly enhancing TNF-α, IL-6 and IL-1β production in THP-1 monocytes and TNF-α production in THP-1-derived macrophages in a dose-dependent manner. In addition, PP crude extract promoted IL-10 production in human monocytes and macrophages with less impact. These results are consistent with previous studies showing that mushroom crude extract and crude polysaccharides had both inflammatory and anti-inflammatory effects, including TNF-α, IL-1β, IL-6, and IL-10 production [28, 29]. These proinflammatory cytokines are required for dendritic cell activation and Th1-mediated immunity [30]. Therefore, the findings underscore the potential immune-boosting advantages of PP as a natural supplement for enhancing immune response, especially in monocytes and macrophages of immunocompromised individuals.

The absence of vaccines for *C. neoformans* prevention is a significant factor contributing to life-threatening infection and increased morbidity and mortality rates worldwide [31]. Therefore, discovering the strategies to empower immune cells when encountering *C. neoformans* infection is an urgent challenge. Previous studies demonstrated the effect of mushroom β-glucan and chitin from *Saccharomyces cerevisiae* to enhance monocyte responses to subsequent bacterial infection [10, 32]. This phenomenon is termed trained immunity. Trained immunity or innate immune memory is a long-term functional reprogramming of the innate immune cells, which provides long-lasting protection to unrelated infectious agents. Monocytes or macrophages recognize training agents via PRRs, which initiate a cascade of events, resulting in cellular metabolic changes, epigenetic reprogramming, and histone modifications. This increases proinflammatory cytokine secretion, including IL-6, IL-1β, TNF-α, and ROS production against subsequent insults [19, 33, 34]. Trained immunity provides key advantages by improving the efficiency of the innate immune response to secondary stimuli. As a result, the immune system can respond swiftly and robustly, which contributes to effectively controlling infection and reducing disease severity [21]. Recent studies revealed the potential induction of trained immunity by natural β-glucan on monocytes and macrophages against *Mycobacterium tuberculosis* and *Leishmania braziliensis* [33, 34]*.* This study developed a preventive model for *C. neoformans* infection in human THP-1 monocytes and THP-1-derived macrophages, with some modifications from previous models [18, 19].

THP-1 cells were pre-exposed to PP crude extract for 24 h (training state). The PP-treated cells, referred to as “trained cells,” were then rested in complete media for two days to revert to a steady state. Subsequently, PP-trained cells were challenged with a secondary stimulus, which is *C. neoformans* (MOI 1:5). Interestingly, pre-exposure to PP crude extract, particularly 500 µg/mL, promoted pronounced proinflammatory cytokine gene expression in THP-1 monocytes after *C. neoformans* restimulation. The differences in the cytokine pattern in response to secondary stimuli were noted. Restimulation with LPS, commonly used as a secondary stimulus in the trained immunity model, induced *TNFA*, *IL1B,* and *IL6* expression [18]. In contrast, restimulation with opsonized *C. neoformans* in PP-trained THP-1 monocytes.primarily induced *IL1B* and *IL6* expression. These findings suggest that the immunological response pattern is influenced not only by the type and intensity of the initial exposure [35], but also by the nature of the secondary stimulus. This is supported by previous studies showing that training primary monocytes with *Saccharomyces cerevisiae* enhanced proinflammatory cytokine production upon secondary stimulation with LPS or Pam3CSK4, but not in response to *Candida albicans.* [32]. In addition, *C. neoformans* possesses numerous virulence factors that suppress proinflammatory cytokine production [36] while simultaneously promoting anti-inflammatory cytokine responses [37]. This may explain why the expression levels of proinflammatory cytokines were lower compared to those observed in macrophages restimulated with LPS. Furthermore, our findings indicate that monocytes pre-exposed to PP and subsequently differentiated into macrophages displayed a significant increase in *TNFA* gene expression and a decrease in *IL10* gene expression when infected with *C. neoformans*, compared to the non-trained group. This suggests that the functional reprogram induced by PP is retained through the cell differentiation process, highlighting the potential for a long-lasting protective effect of PP crude extract in pre-exposed or trained cells.

*C. neoformans* virulence factors directly induced pathogenic M2 polarization while suppressing the *MCP1/CCL2* M1 marker [38]. Moreover, *C. neoformans* infection induced a Type-2 non-protective immune response and M1 repolarization to the M2 phenotype [39, 40]. β-glucan has been documented to induce protective trained immunity by histone modification in the regulatory region of the *MCP1/CCL2* gene and promote *MCP1/CCL2* expression against *Mycobacterium tuberculosis* infection [34]. Hence, we explored the effect of PP crude extract on macrophage polarization after *C. neoformans* infection. PP crude extract was utilized as a training stimulus in THP-1 monocytes before inducing macrophage differentiation. Following this, M1/M2 polarization in *C. neoformans*-infected macrophages was investigated. Compared with the M0 macrophage, the *MCP1/CCL2* expression level was significantly downregulated, while the *TGM2* expression level was slightly upregulated in non-trained macrophages infected with *C. neoformans.* The results indicate that *C. neoformans* influenced macrophage polarization by suppressing the M1 phenotype. Interestingly, higher levels of *MCP1/CCL2* and lower *TGM2* gene expression were observed in the infected macrophages pre-exposed to PP crude extract and β-glucan (data not shown). The significant upregulation of *MCP1/CCL2* is vital in the inflammation process and could boost antifungal immune response. MCP1/CCL2 promoted the recruitment and activation of monocytes and macrophages during inflammation and modulated Th1 immune response through MCP1/CCL2-CCR2 interaction [41–43]. Furthermore, pre-exposure to PP crude extract appeared to suppress CD206 expression in *C. neoformans*-infected macrophages, both in the presence and absence of the Th2 cytokine IL-4. The findings suggest that PP crude extract promotes M1 polarization while suppressing M2 differentiation during *C. neoformans* infection, potentially in part through a β-glucan-mediated mechanism.

Intracellular proliferation within phagocytes is a key factor in *C. neoformans* pathogenesis, enabling its dissemination to the central nervous system [8]. Our findings demonstrate that pre-exposure to 100 µg/mL of PP crude extract significantly reduced the intracellular proliferation of *C. neoformans* to 58% in trained macrophages compared to non-trained controls. This reduction in fungal burden reflects enhanced intracellular fungal clearance, suggesting that trained macrophages are more effective at restricting the growth of *C. neoformans*. This effect was also associated with a significant increase in ROS generation in the PP-pretreated group. Given the essential role of ROS in mediating acute inflammation, promoting M1 polarization, and facilitating *C. neoformans* clearance [44, 45], our results suggest that PP crude extract enhances M1 polarization and promotes fungal killing, at least in part, through ROS production. Collectively, these findings indicate that pre-exposure to 100 µg/mL of PP crude extract induces a trained macrophage phenotype that enhances fungal elimination and strengthens host defense against *C. neoformans* infection.

Previous studies have shown that crude polysaccharides from *P. pulmonarius*, particularly β-glucan-rich fractions, modulate immune responses by upregulating *TNFA*, *IL1B*, and *IL6* gene expression in THP-1-derived macrophages [46]. Given these findings, β-glucans are likely major contributors to the immunostimulatory effects observed in our study. However, LC–MS/MS analysis of *P. pulmonarius* crude extract identified additional bioactive compounds, including diacylglycerol, glutathione, a dihydroresveratrol derivative, and aspartic acid [53]. These compounds have been associated with type 1 immune responses, M1 macrophage polarization, and trained immunity [47–52], suggesting that the observed effects may result from synergistic interactions rather than a single active component. While β-glucan is a well-established immunomodulator, our findings indicate that other constituents in the extract may also contribute to macrophage activation and antifungal defense. To delineate the specific contributions of β-glucan, future studies should focus on its purification and direct comparison with the crude extract to distinguish its isolated effects from potential synergistic interactions with other bioactive compounds.

Given its immunomodulatory properties, PP crude extract holds promise as a prophylactic or adjunctive therapy against* C. neoformans* infections, particularly in immunocompromised individuals, such as those living with HIV/AIDS or receiving immunosuppressive therapy. To support its clinical potential, future studies should assess its role in trained immunity using an in vivo priming model and explore potential synergistic effects with standard antifungal agents to improve therapeutic efficacy, especially in high-risk populations. With further research, PP crude extract may emerge as a novel immunotherapeutic strategy that complements conventional antifungal treatments and strengthens host defense against *C. neoformans* infections.

## Conclusions

**Fig. 6:**
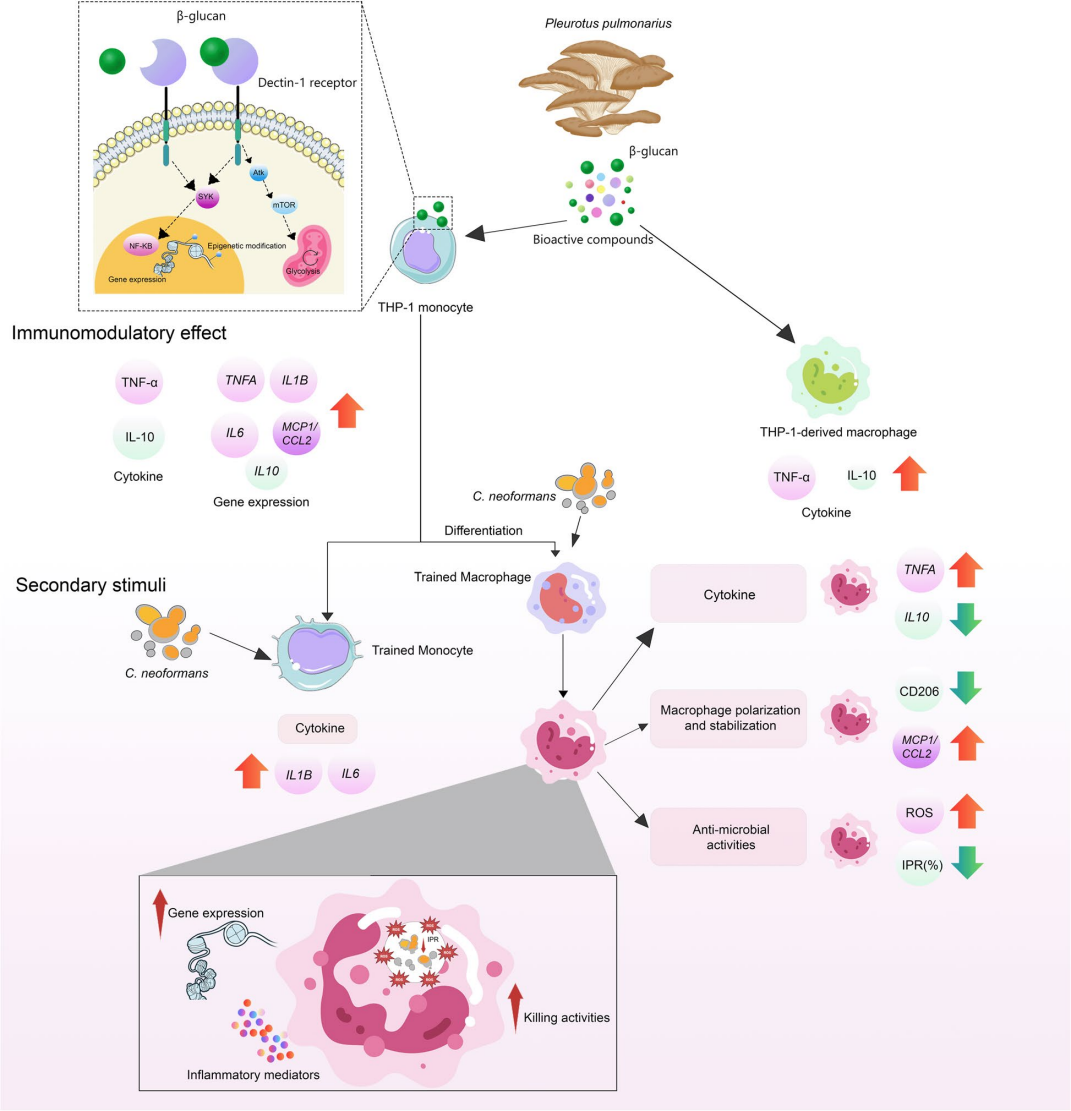
Schematic representation of the immunomodulatory effects of *Pleurotus pulmonarius* (PP) crude extract on THP-1 monocytes and THP-1-derived macrophages in response to *C. neoformans*. Dashed lines represent hypothetical mechanisms based on existing literature. We proposed that the PP crude extract primarily exerts its effects through β-glucan-Dectin-1 interaction, leading to activation of the Syk-NF-κB signaling cascade. This activation enhances the production of proinflammatory cytokines (IL-6, IL-8, and IL-1β) and increases ROS generation, both of which are essential for effective fungal clearance. Furthermore, the β–glucan-Dectin-1 signaling may induce a trained immunity phenotype, thereby priming innate immune cells for an augmented response upon subsequent encounters

This study demonstrates the dose-dependent immunomodulatory effects of *Pleurotus pulmonarius* (PP) crude extract on THP-1 monocytes and macrophages, proposing potential antifungal mechanisms (illustrated in Fig. 6). The PP crude extract enhances proinflammatory cytokine production, promotes M1 macrophage polarization, suppresses M2 polarization, increases ROS production, and exhibits antifungal activity against *C. neoformans*. These findings establish PP crude extract as a promising natural immunomodulator that strengthens innate immunity and enhances host defense, particularly in immunocompromised individuals. Furthermore, this study highlights its potential applications of PP crude extract in alternative antifungal therapies, functional foods, and trained immunity-based interventions. By demonstrating its immunomodulatory efficacy, this research lays the foundation for future investigations into the preventive and therapeutic potential of PP against microbial infections.

## Data Availability

All data analyzed during this study are included in this published article. Additional datasets generated and/or analyzed during the current study are available from the corresponding author upon reasonable request.

## Abbreviations

*C. neoformans*: *Cryptococcus neoformans*
CFU: Colony-forming unit
IL10: Interleukin-10
IL1B: Interleukin-1 beta
IL6: Interleukin-6
IPR: Intracellular proliferation rate
LPS: Lipopolysaccharide
MCP1/CCL2: Monocyte chemoattractant protein-1
MOI: Multiplicity of infection
MTT: 3-(4,5-Dimethyl-2-thiazolyl)-2,5-diphenyl-2H-tetrazolium bromide
NT: Non-trained
PBS: Phosphate-buffered saline
PMA: Phorbol 12-myristate 13-acetate
PP: *Pleurotus pulmonarius*
RT-qPCR: Quantitative reverse transcription PCR
rhIFN-γ: Recombinant interferon gamma
rhIL4: Recombinant interleukin-4
ROS: Reactive oxygen species
RPMI: Roswell Park Memorial Institute 1640 Medium
SDA: Sabouraud dextrose agar
SDS: Sodium Dodecyl Sulfate
TGM2: Transglutaminase 2
TLRs: Toll-like receptors
TNFA: Tumor necrosis factor alpha
TNF-α: Tumor necrosis factor alpha
